# Structural basis for biologically relevant mechanical stiffening of a virus capsid by cavity-creating or spacefilling mutations

**DOI:** 10.1038/s41598-017-04345-w

**Published:** 2017-06-22

**Authors:** Pablo Guerra, Alejandro Valbuena, Jordi Querol-Audí, Cristina Silva, Milagros Castellanos, Alicia Rodríguez-Huete, Damià Garriga, Mauricio G. Mateu, Nuria Verdaguer

**Affiliations:** 1 Structural Biology Unit, Institut de Biologia Molecular de Barcelona (CSIC). Parc Científic de Barcelona, Baldiri i Reixac 15, E-08028 Barcelona, Spain; 2 Centro de Biología Molecular “Severo Ochoa” (CSIC-UAM), Universidad Autónoma de Madrid, Madrid, 28049 Spain; 3 Infection and Immunity Program and Department of Biochemistry and Molecular Biology, Biomedicine Discovery Institute, Monash University, Clayton, Victoria 3800 Australia

## Abstract

Recent studies reveal that the mechanical properties of virus particles may have been shaped by evolution to facilitate virus survival. Manipulation of the mechanical behavior of virus capsids is leading to a better understanding of viral infection, and to the development of virus-based nanoparticles with improved mechanical properties for nanotechnological applications. In the minute virus of mice (MVM), deleterious mutations around capsid pores involved in infection-related translocation events invariably increased local mechanical stiffness and interfered with pore-associated dynamics. To provide atomic-resolution insights into biologically relevant changes in virus capsid mechanics, we have determined by X-ray crystallography the structural effects of deleterious, mechanically stiffening mutations around the capsid pores. Data show that the cavity-creating N170A mutation at the pore wall does not induce any dramatic structural change around the pores, but instead generates subtle rearrangements that propagate throughout the capsid, resulting in a more compact, less flexible structure. Analysis of the spacefilling L172W mutation revealed the same relationship between increased stiffness and compacted capsid structure. Implications for understanding connections between virus mechanics, structure, dynamics and infectivity, and for engineering modified virus-based nanoparticles, are discussed.

## Introduction

The study of viruses and their protein capsids using single-molecule techniques and theoretical physicochemical approaches is leading to a physics-based understanding of different aspects of virus biology^1–13^. In particular, atomic force microscopy (AFM) is being used to experimentally determine the mechanical properties of virions and their protein capsids^4, 7, 11, 14, 15^. Virus particles of different lineages have been found to differ widely in stiffness, brittleness, resistance to mechanical disruption and materials fatigue^6^; and analysis of purposefully modified virions and capsids supports different functional roles for virus mechanics^3, 4, 16–23^. The behavior of viruses under mechanical force appears to have been genetically shaped during biological evolution because of their adaptive value.

Modeling and coarse-grained molecular dynamics (MD) simulations are being used to investigate structural features underlying the mechanical response of virus capsids of different species (reviewed in refs 4, 8 and 10). Virus components such as capsid proteins and nucleic acid, and specific capsid amino acid side chains are being identified by AFM as determinants of virus mechanics^16–21, 24–30^. Based on these developments, understanding viral function in mechanical terms could greatly benefit from all-atom structural studies of variant viruses of a same species in which single point mutations modify biological function through changes in mechanical properties. All-atom MD is still difficult to apply for this goal because of the large size of viral complexes^10^, but MD-based multiscale modeling studies are being used to probe atomic-level details of virus mechanics^31, 32^. In addition, comparing atomic structures solved by X-ray crystallography or by high-resolution cryo-electron microscopy provides an excellent experimental approach for such purpose. Understanding capsid structure-mechanics relationships in atomic detail may also facilitate the rational engineering of mechanically improved virus nanoparticles for technological applications^33–39^.

**Figure 1:**
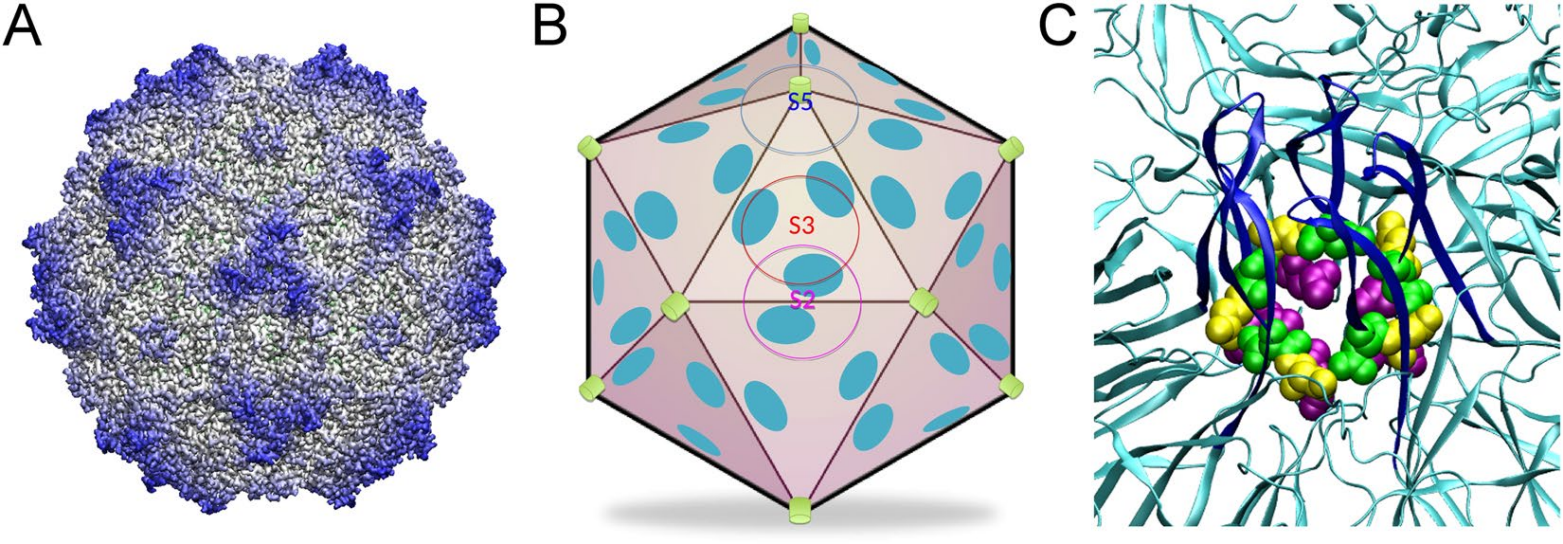
The MVM capsid. (**A**) Crystal structure of the wt MVMp capsid (PDB id 1Z14)^41^ represented as a surface model. (**B**) Scheme of the MVM capsid architecture. Orientation is approximately the same as in panel (**A**). One symmetry axis of each type (S5, S3, S2) is labeled. Capsid pores at the S5 axes are indicated by green cylinders, and inner sites where DNA segments are bound are shown as cyan ellipses. (**C**) Close-up of the region around a pore in the MVMp capsid, represented as a cartoon model. The β-cylinder delimiting the pore is colored deep blue. Amino acid residues N170, D171 and L172 corresponding to each of the five capsid subunits surrounding the pore are represented as spacefilling models and respectively colored green, yellow or purple.

We have been using the parvovirus minute virus of mice (MVM) as a model for studies on virus biomechanics. The MVM virion is one of the smallest (25 nm in diameter) and structurally simplest known^40^, which facilitates structure-properties-function analyses in atomic detail. The virion (Fig. 1A)^41^ is built from a self-assembled T = 1 icosahedral capsid (Fig. 1B)^42, 43^ into which the single-stranded (ss) DNA viral genome is later packaged^44^. The capsid is made of 60 protein subunits (VPs) with identical sequence and fold, except for their N-terminal segments (Nts). The Nts, longer in 10 (VP1) subunits than in the other 50 (VP2) subunits, are structurally disordered, initially located inside the capsid, and do not contribute to capsid structure^41, 43^, but carry signals important for viral infection^45, 46^. Pores (channels) at the capsid 5-fold symmetry (S5) axes (Fig. 1B) are involved in DNA encapsidation^44^, uncoating^46^, and biologically relevant translocation of VP2 and VP1 Nts in the virion^42, 46–52^. Segments of the viral ssDNA are folded as “wedges” that bind concavities at the capsid inner wall, close to the 2-fold (S2) axes and farthest from the pores at the S5 axes (Fig. 1B)^41^.

In previous biomechanical studies we used MVM (strain p, which infects fibroblasts). We found that capsid-bound ssDNA segments determine an anisotropic distribution of stiffness in the virion by locally stiffening S2 regions, but not distant S5 (pore) regions^18, 25^. Mutations of capsid residues that specifically eliminated some interactions between the capsid and the bound DNA segments softened S2 regions in the virion, and also reduced the resistance of the virion against heat-induced inactivation^18, 25^. In contrast, mutation of amino acid residues located at the base of the pores (e.g., N170A) invariably led to stiffening of the S5 (pore) regions in the capsid^19^, and were deleterious for viral infection^53^. The results revealed that the distribution of stiffness in MVM provides the biological advantage of optimizing virion resistance against thermal inactivation of infectivity, without altering the conformational dynamism of the capsid pore regions that is required for infection^19, 20^.

From a structural point of view it was remarkable that, unlike holes in a sponge, the creation of cavities in the MVM capsid by individually removing different chemical groups around the capsid pores invariably led to a large increase, and not a decrease, in mechanical stiffness of the pore regions^19^. This stiffening was inextricably linked to the impairment of a subtle capsid conformational transition^52, 53^ associated to translocation through the pores of functionally important signals carried by VP2 Nts^49^. Impaired externalization of Nts contributed to explain the deleterious effect of these mutations in the virion, which lost its infectivity at physiological temperature^53^. Mutant virions were not formed and could not be analyzed; however, this biologically relevant externalization event and its impairment by mutation could be specifically studied *in vitro* using VP2-only capsids subjected to moderate heating, which contributed the energy needed to trigger the conformational change^52^.

As a part of a different study on MVM biology using a different viral strain (strain i, which infects T-lymphocytes), other researchers showed that the spacefilling mutation L172W, also located at the base of the capsid pores, was detrimental for viral infectivity^54^. Determination of the X-ray structure of the L172W mutant capsid indicated that the functional effect of this mutation was related to a reduction in the diameter of the capsid pores, which prevented DNA packaging^44^. The effect of this mutation on capsid stiffness was not analyzed.

The above findings together provided an excellent case study to experimentally investigate the relationship between atomic structure and biologically relevant mechanical properties of a virus particle. Here we have compared the effects on mechanical stiffness and atomic structure of the MVM capsid of two biologically relevant mutations at the capsid pore wall: the cavity-creating N170A^19, 53^ and the spacefilling L172W^44, 54^ (Fig. 1C). Both N170A (in strain p) and L172W (in strain i) individually caused multiple phenotypic defects, including either unconditional or temperature-sensitive deficiencies in capsid assembly, genome packaging, uncoating that occurred prematurely, externalization of Nts and/or entry into cells^46, 53–55^; however, their virus-inactivating effects were traced to different causes.

We have now determined the crystal structure at 3.8 Å resolution of the mutant N170A VP2-only capsid, and compared it with the crystal structure of the non-mutated VP2-only capsid (strain p). The results reveal that the capsid-stiffening, cavity-creating N170A mutation neither modifies the diameter of the pore nor induces any dramatic conformational change around the pores; instead, it generates subtle rearrangements that propagate through the whole capsid, resulting in a more compact, less flexible structure. We have also determined that the spacefilling L172W mutation also stiffens the MVM capsid (strain i). Structural comparison indicates that the L172W-mediated stiffening effect is also related to a more compact capsid structure. We discuss the implications for understanding connections between virus structure, dynamics, mechanics and infectivity, and the potential of these studies for nanotechnological applications.

## Results and Discussion

### Compared local and global mechanical effects of cavity-creating N170A and spacefilling L172W mutations in the MVM capsid

In order to undertake a detailed experimental comparison between MVM capsid mechanics and atomic structure, we used the VP2-only capsids of viral strains p and i. Use of VP2-only capsids allowed us to avoid some ambiguities when interpreting structure-properties-function relationships. Use of two different MVM strains was determined by the fact that different strains had been previously used to investigate biological and other effects of these mutations. Investigation using mutant virus was prevented because of the deleterious effects of the mutations of interest (N170A and L172W).

**Figure 2:**
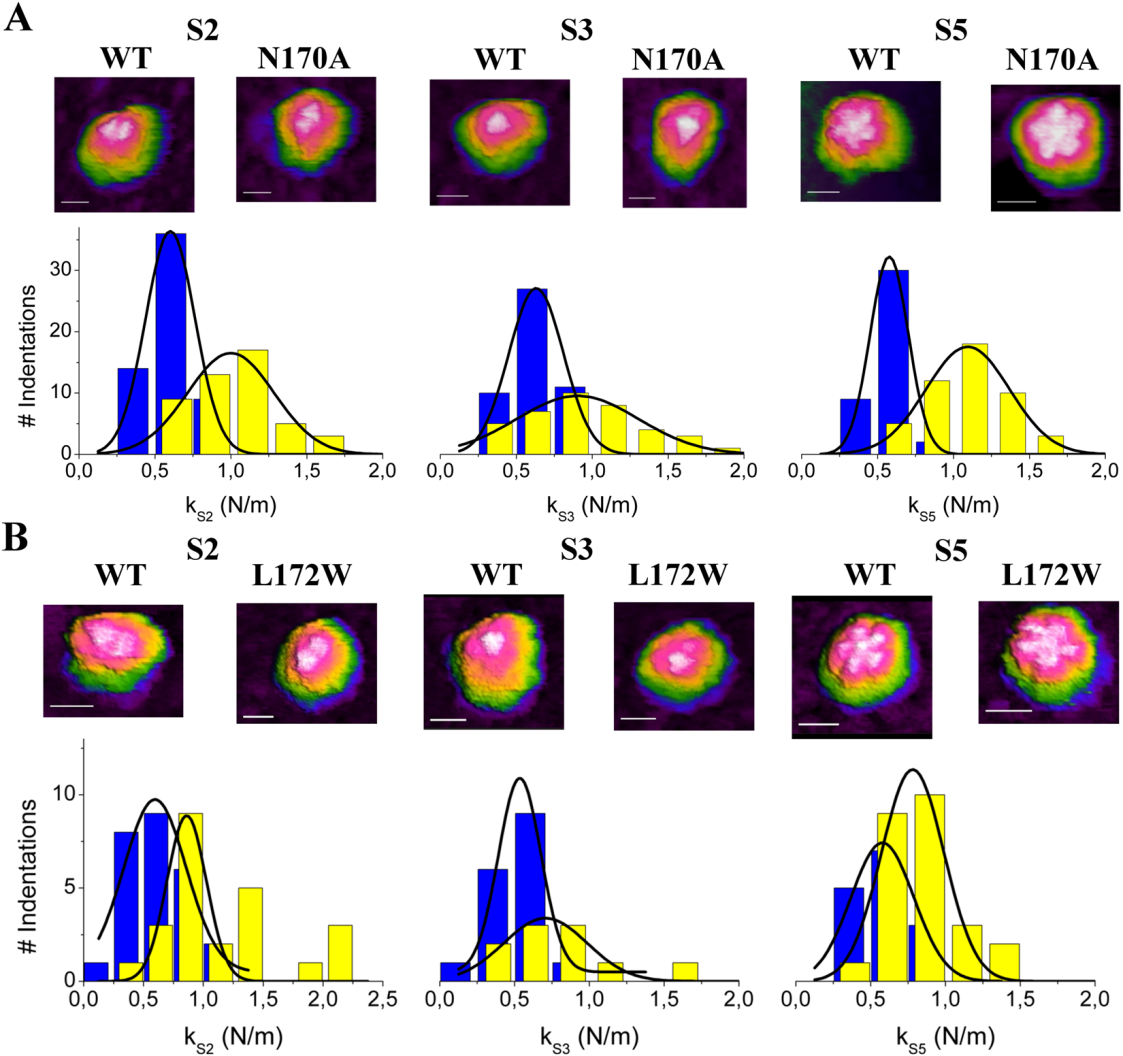
Topography and mechanical stiffness of wt and mutant MVM capsids. (**A**) N170A mutant and wt capsids of MVMp. (**B**) L172W mutant and wt capsids of MVMi. In each panel, AFM images of individual capsids respectively oriented along a S2 (left), S3 (middle) or S5 (right) axis are depicted on top. Scale bar in images is 15 nm in length. Below the images, elastic constant *k*_s_ distributions determined by AFM for capsids oriented along different symmetry axis are shown. Each histogram represents the number of individual determinations *versus* the *k*_s_ obtained for the mutant capsid (yellow bars) and the wt capsid (blue bars) along a S2 (left plot), S3 (middle) or S5 (right) axis in the capsid. Data were fitted to Gaussian distributions. See Table 1 for average values and statistical analysis.

First, we used AFM to determine the individual effects on stiffness of the VP2-only MVMp capsid caused by the deleterious, cavity-creating N170A mutation in strain p. Sequencing of the entire capsid-coding region confirmed that the primary structures of the wt and N170A mutant capsids used differ exclusively in residue 170 (asparagine or alanine, respectively) (Fig. S1 of Supporting Information, SI). Both capsids were expressed and self-assembled in cells and purified. For each single capsid analyzed, AFM imaging in physiological buffer (phosphate-buffered saline, PBS) revealed the correct particle height. It also allowed the visualization of major topographic features located at different symmetry axes (wide and tall “spikes” at S3, protruding “cylinders” at S5 and depressions at S2 axes; Fig. 2, and Fig. S2 of SI). By identifying these structural features, the orientation of each individual particle could be ascertained, and the approximate region indented during mechanical analysis of the imaged particle could be identified (Fig. 2, and Fig. S2 of SI)^18, 19, 25^.

The elastic constants *k*_s_ of the N170A VP2-only capsid at regions centered at S5, S3 or S2 axes were then determined, and compared with those of the wt VP2-only capsid. The AFM tip was used to indent intact, adequately oriented individual viral particles in PBS. For each capsid region, many valid force *versus* z-displacement (F-Z) curves were obtained using several particles (see Experimental section and Fig. S3 in SI). F-Z curves (Fig. S3D) were converted into force-indentation (particle deformation) (F-D) curves (Fig. S3E). For each indentation event, the data were used to determine: i) *k*_s_ values during the elastic deformation of the viral particle (after the weakly non elastic deformation that occurred at the beginning of the indentation); and ii) maximum particle deformation achieved, to make sure it was small enough (<2 nm) to keep the particle under the elastic deformation regime. For each individual particle analyzed several indentations were carried out, and several particles were used to determine stiffness for each particle type in each orientation (a S2, S3 or S5 axis on top), as detailed in the Experimental section. Under the indentation conditions used (including maximum force and time between serial indentations), no signs of mechanical fatigue were detected for the particles analyzed. AFM imaging showed that each particle recovered its original height and topography after indentation (compare Fig. S3A and S3B in SI), and no trend was observed when *k*_s_ values obtained in serial indentations were compared (Fig. S4 in SI). MD simulations indicate that stiffness may depend on indentation depth because of structural remodeling^31^. However, no significant variation in *k*_*s*_ as a function of indentation depth was observed, perhaps because any difference was within the experimental errors of our measurements.

For different particle types (wt and mutant) and region indented, the average *k*_s_ values were compared and the differences between them were statistically validated. The results (Fig. 2A and Table 1) showed that the N170A single mutation caused statistically significant, large increases in stiffness of the VP2-only capsid (80% at S5 regions, 50% at S3 regions and 59% at S2 regions). Comparison with results obtained using VP1/VP2 MVMp capsids^19^ revealed that the capsid stiffening effect of the N170A mutation does not depend on the presence or absence of the structurally disordered VP1 Nt extensions (Table 1).

**Table 1:** Elastic constant *k*_s_ values^a^ determined for MVM capsids (wt and N170A and L172W mutants) along S2, S3 or S5 axes.

MVM capsid^b^	S2				S3				S5			
	*k*_*s*_ (N/m)^c^	*Fz* ^d^	*n* ^e^	*p* ^f^	*k*_*s*_ (N/m)^c^	*Fz* ^d^	*n* ^e^	*p* ^f^	*k*_*s*_ (N/m)^c^	*Fz* ^d^	*n* ^e^	*p* ^f^
**wtp**_**VP1/VP2**_	**0.55 ± 0.14**	109	14	—	**0.54 ± 0.14**	110	16	—	**0.59 ± 0.11**	99	6	—
**wtp**_**VP2**_	**0.63 ± 0.12**	41	7	0.0009	**0.62 ± 0.16**	52	10	0.002	**0.61 ± 0.11**	24	4	0.59
**wti**_**VP2**_	**0.62 ± 0.27**	26	6	0.064	**0.52 ± 0.17**	17	8	0.64	**0.61 ± 0.14**	15	2	0.48
**N170Ap**_**VP1/VP2**_	**1.00 ± 0.48**	99	12	3 × 10^−18^	**0.88 ± 0.35**	113	20	8 × 10^−18^	**0.98 ± 0.21**	123	5	1 × 10^−40^
**N170Ap**_**VP2**_	**1.00 ± 0.27**	46	8	6 × 10^−30^	**0.93 ± 0.37**	38	10	2 × 10^−16^	**1.10 ± 0.25**	48	8	5 × 10^−36^
**L172Wi**_**VP2**_	**1.16 ± 0.51**	24	7	7 × 10^−20^	**0.82 ± 0.35**	10	4	3 × 10^−6^	**0.82 ± 0.24**	25	6	7 × 10^−11^

^a^k_s_ values for wtp_VP1/VP2_ and N170Ap_VP1/VP2_ have been previously published and are included here for completeness.

^b^p, strain p; i, strain i; _VP1/VP2_, capsid containing 10 VP1 and 50 VP2 subunits; _VP2_, capsid containing 60 VP2 subunits.

^c^Elastic constant (average ± standard deviation).

^d^Number of indentations used for analysis.

^e^Number of individual particles used for analysis.

^f^ *p*-value relative to wtp_VP1/VP2_, obtained in a Student t-test with an alpha level = 0.05.

We then likewise analyzed the effect on stiffness of the VP2-only MVMi capsid caused by the detrimental, spacefilling L172W mutation. The appropriate recombinant plasmids were constructed, and sequencing confirmed that the primary structures of strain i wt and L172W mutant capsids differ exclusively in residue 172 (leucine or tryptophan, respectively). The wt and L172W MVMi capsids were expressed and assembled in cells and purified, and their elastic constants *k*_s_ were determined exactly as carried out for the wt and N170A MVMp capsids. The results (Fig. 2B and Table 1) showed that the mechanical stiffness of S2, S3 and S5 regions were virtually indistinguishable between wt capsids of viral strains p or i (Table 1), even though they differ in 13 amino acid residues per subunit (780 residues in total). In contrast, the single mutation L172W, like the N170A mutation, caused quite significant, large increases in *k*_s_ (35% at S5 regions, 58% at S3 regions and 87% at S2 regions; Fig. 2B and Table 1).

In this and previous studies we generally observed that the higher the average *k*_*s*_ determined, the wider the distribution of individual *k*_*s*_ values, even when well-structured regions of a same viral particle were compared. A typical example was provided by the MVM virion: indentations along a S2 region yielded both a higher average *k*_*s*_ value and a wider distribution of values compared to indentations along a S5 region^25^. This association between higher stiffness an wider dispersion of *k*_*s*_ values may be related with some side effect during indentation of the higher force required to deform a stiffer region (to be investigated).

To sum up, multiple amino acid differences in the capsid between two natural MVM strains (as many as 13 residues per capsid subunit) did not cause any significant difference in mechanical stiffness. In contrast, individual mutation of neighboring amino acid residues lining the wall of the capsid pores caused large increases in stiffness. These mutations included a cavity-creating mutation (N170A) in strain p, and a spacefilling mutation (L172W) in strain i, which were both detrimental for MVM infectivity through different biological mechanisms. The N170A mutation had a larger local (S5 regions) stiffening effect, while the L172W mutation caused a greater stiffening of capsid regions (S2) distant from the pores.

### Compared effects on capsid conformational dynamics of N170A and L172W mutations

We had previously observed that the mechanically rigidifying, cavity-creating N170A mutation impairs a biologically relevant conformational rearrangement of the MVMp capsid^53^. This structural transition was analyzed *in vitro* using the VP2-only MVMp capsid to avoid some ambiguities in interpretation that could arise if VP1 Nts were present, and also because mutant virions could not be produced because of the deleterious effect of this mutation. The transition was triggered *in vitro* by moderate heating, and detected as a subtle but entirely reproducible change in fluorescence of some capsid tryptophans^52^ (see the Introduction and Fig. S5A in SI) and a change in capsid equilibrium dynamics^56^. We have now tested, again using spectrofluorimetry, whether an equivalent transition also occurs in the VP2-only capsid of a different MVM strain (strain i), and whether the L172W mutation, like the N170A mutation, could impair this conformational rearrangement. The results (Fig. S5B in SI) revealed that the transition does take place in the wt MVMi capsid, but not in the L172W mutant. The somewhat different transition temperature of  the two wt capsids (47.2 ± 0.3 °C versus 54 ± 2 °C) is likely due to the 13-residue difference between them. To sum up, both MVM strains i and p undergo a conformational rearrangement that had been previously associated to VP2 Nt externalization; and both N170A and L172W mutations of residues delineating the wall of the capsid pores impair MVM capsid dynamics related to biologically relevant through-pore translocation events.

### Crystal structure of a mechanically stiffened N170A mutant MVM capsid, and comparison with the non-mutated capsid structure

To investigate in atomic detail the structural basis of the mechanical stiffening caused by the cavity-creating N170A mutation, we determined the crystal structure of the VP2-only capsid of N170A mutant MVMp. Then, we compared it with the crystal structure of the VP2-only capsid of wt MVMp, which had been previously solved at 3.25 Å resolution^43^.

The N170A MVMp capsid crystallized in the rhombohedral space group R32 (Table 2). Packing considerations indicated that the crystal asymmetric unit contained 1/6 or 10 protomers of two different virions, with one of the crystallographic S2 and S3 axes coinciding with one icosahedral S2-fold and S3 axes for each of the virion particles in the unit cell. The structure was solved at 3.8 Å resolution by molecular replacement using 2 × 10-fold non-crystallographic symmetry averaging, starting with the phases corresponding to that of the wt MVMp VP2-only capsid structure (PDB id: 1Z14)^43^. The averaging mask covered the whole asymmetric unit.

**Table 2:** X-ray data collection and refinement statistics for the N170A mutant MVMp capsid.

Data collection	
Space group	R32
**Cell dimensions**	
a, b, c (Å)	410.19, 410.19, 559.7
α, β, γ (°)	90, 90, 120
Resolution (Å)*	49.9–3.8 (3.94–3.8)
R_merge_	0.203 (0.789)
I/σI	7.7 (2.4)
Completeness (%)	99.8 (97.7)
Redundancy	10.5 (9.9)
**Refinement**	
Resolution (Å)	49.9–3.8
No. reflections	345341
^†^R_work_/^‡^R_free_	0.282/0.289
**No. atoms**	
Protein	4314
***B*** **-factors**	
Protein	96.9
**R.m.s. deviations**	
Bond lengths (Å)	0.003
Bond angles (°)	0.64

^†^R_work_ = ∑hkl ||Fobs(hkl)| − |Fcalc(hkl)||/∑hkl |Fobs(hkl)|, where Fobs and Fcalc are the structure factors, deduced from measured intensities and calculated from the model, respectively.

^‡^R_free_ = as for R_work_ but for 5% of the total reflections chosen at random and omitted from refinement.

*Values in the parenthesis are of the highest resolution shell.

**Figure 3:**
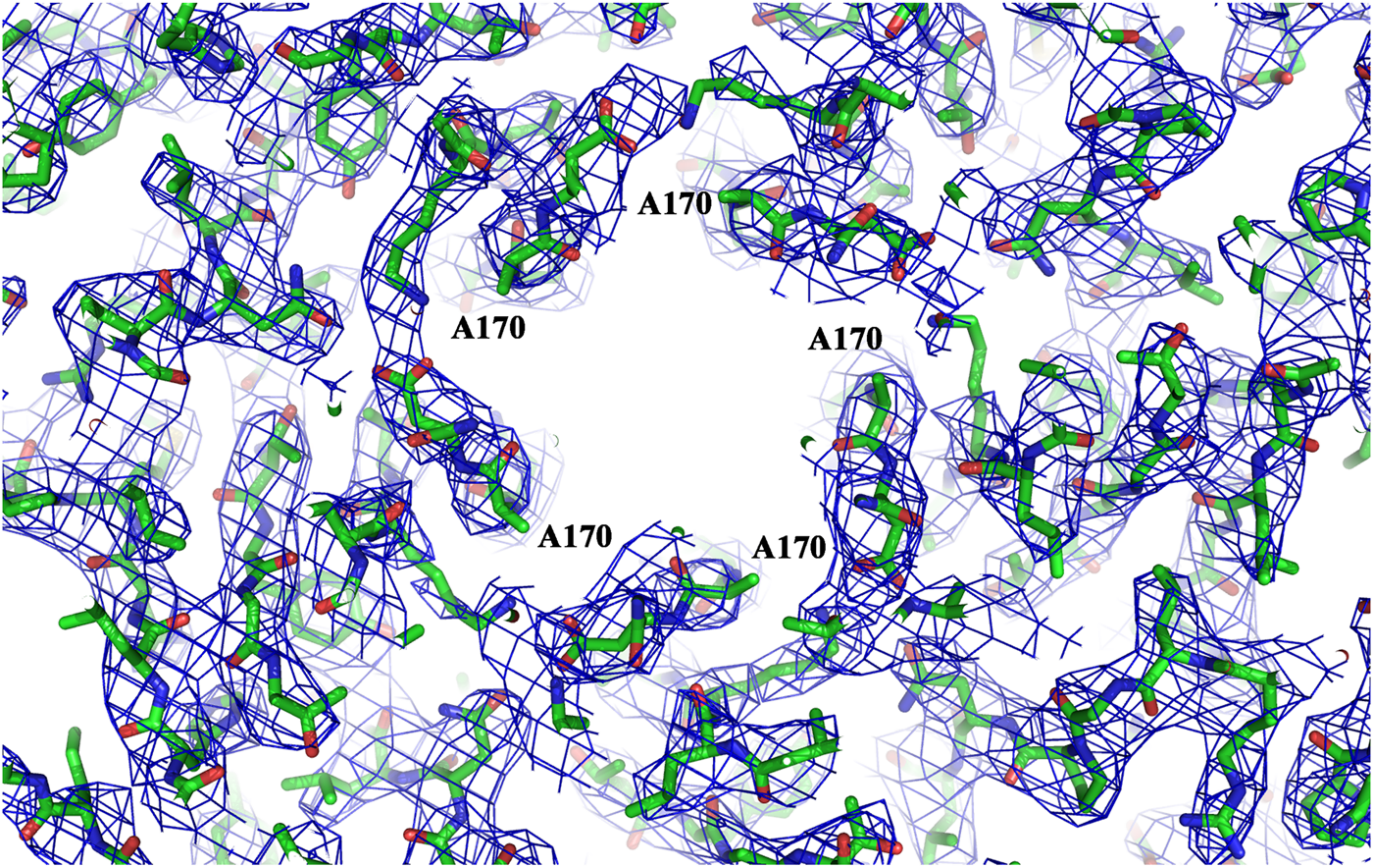
2F_o_–F_c_ averaged electron density corresponding to the N170A mutant MVMp capsid structure (1.5 σ level). The region shown corresponds to the base of the pore at a S5 axis. The substituted residue A170 is explicitly labeled.

The resulting averaged maps showed well-defined density (Fig. 3) which allowed atomic-detailed comparison of subtle structural differences that occurred as a consequence of the mutation. As previously reported for the wt MVMp capsid and other parvovirus capsid structures^41, 43^, the electron density maps were not interpretable for the VP2 Nt (residues 1–38), including the glycine tract within the Nt. In the virion, the Nts have been externalized through the pores and, accordingly, electron density corresponding to the glycine tract is found inside the pores. For the N170A mutant capsid no density was observed within the pores, which indicates that the disordered VP2 Nts are not constitutively externalized.

The structures of N170A and wt capsids are very similar but not identical. The root mean square deviation (RMSD) was 0.58 Å for equivalent Cα atoms in both structures (1.08 Å for all atoms in the 549 VP2 residues superimposed). For comparison, the RMSD between the structures of the wt VP2-only capsid and that of the wt VP1/VP2 capsid (solved at 3.75 Å resolution) was 0.08 Å for equivalent Cα atoms in both structures^43^.

**Figure 4:**
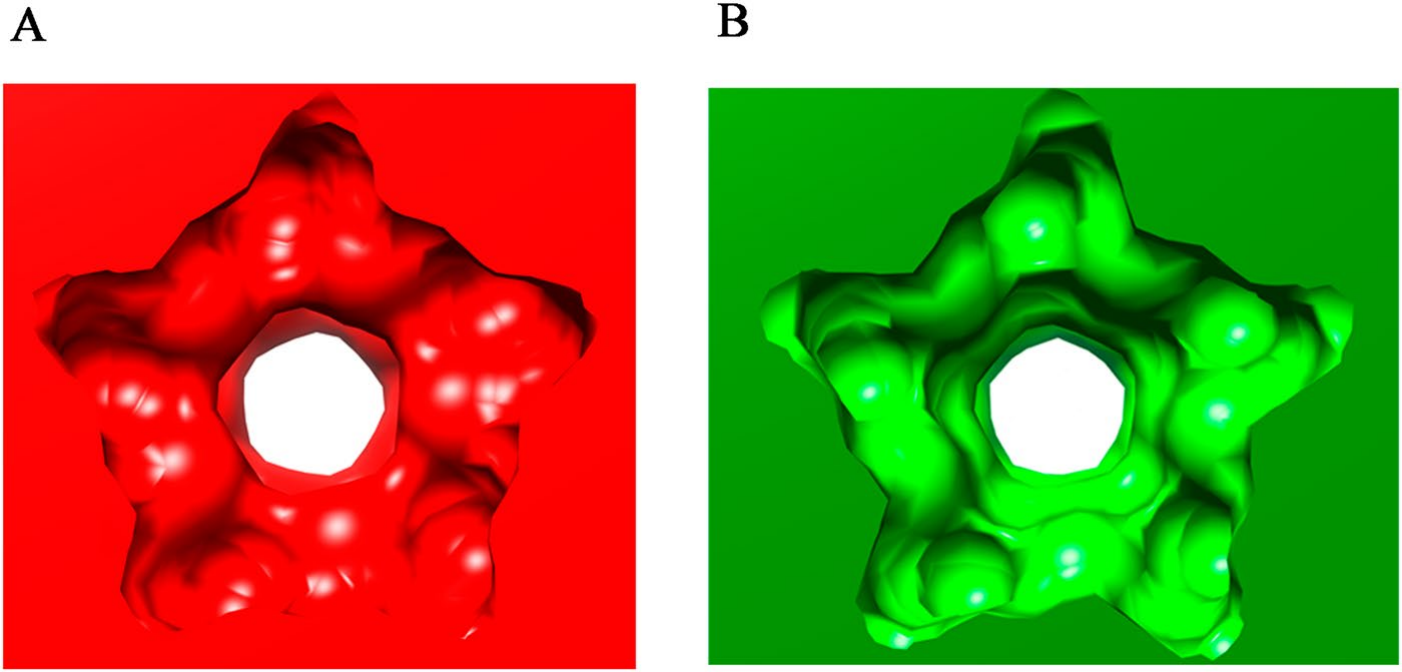
Surface representation of the region around a S5 axis in the MVMp capsid. (**A**) wt capsid (PDB id: 1Z14). (**B**) N170A mutant capsid (this study; PDB id: 4ZPY). View of the inside surface of the capsid. Changes in the apertures of the primary and secondary constrictions along the pore wall (see text) can be observed.

Subtle conformational differences relative to wt were observed both in the vicinity of the mutated residue and elsewhere in the mutant capsid structure. In the ring of five N170/D171 residues located near the base of the β-cylinder that surrounds each capsid pore (Fig. 1C), the N170 amide group can form a hydrogen-bond with the carboxylic group of D171 of the neighboring VP2 subunit. This interprotein interaction cannot occur in the N170A capsid structure. In addition, removal of the amide groups of the five N170 residues generated five cavities around the pore walls (Fig. 4). Slight rearrangements of D171 and T173 side chains, moving towards the new cavity were also observed as a consequence of the mutation. However, the free aperture of the pore at the tightest constriction formed by the ring of L172 residues immediately below the N170/D171 ring (Fig. 1C) remained nearly unchanged, with a diameter of 7.9 Å in the N170A capsid compared to 8.2 Å in the wt capsid (Fig. 4). These values were obtained by measuring the distance between the Cδ atoms of the residues across the capsid pore.

**Figure 5:**
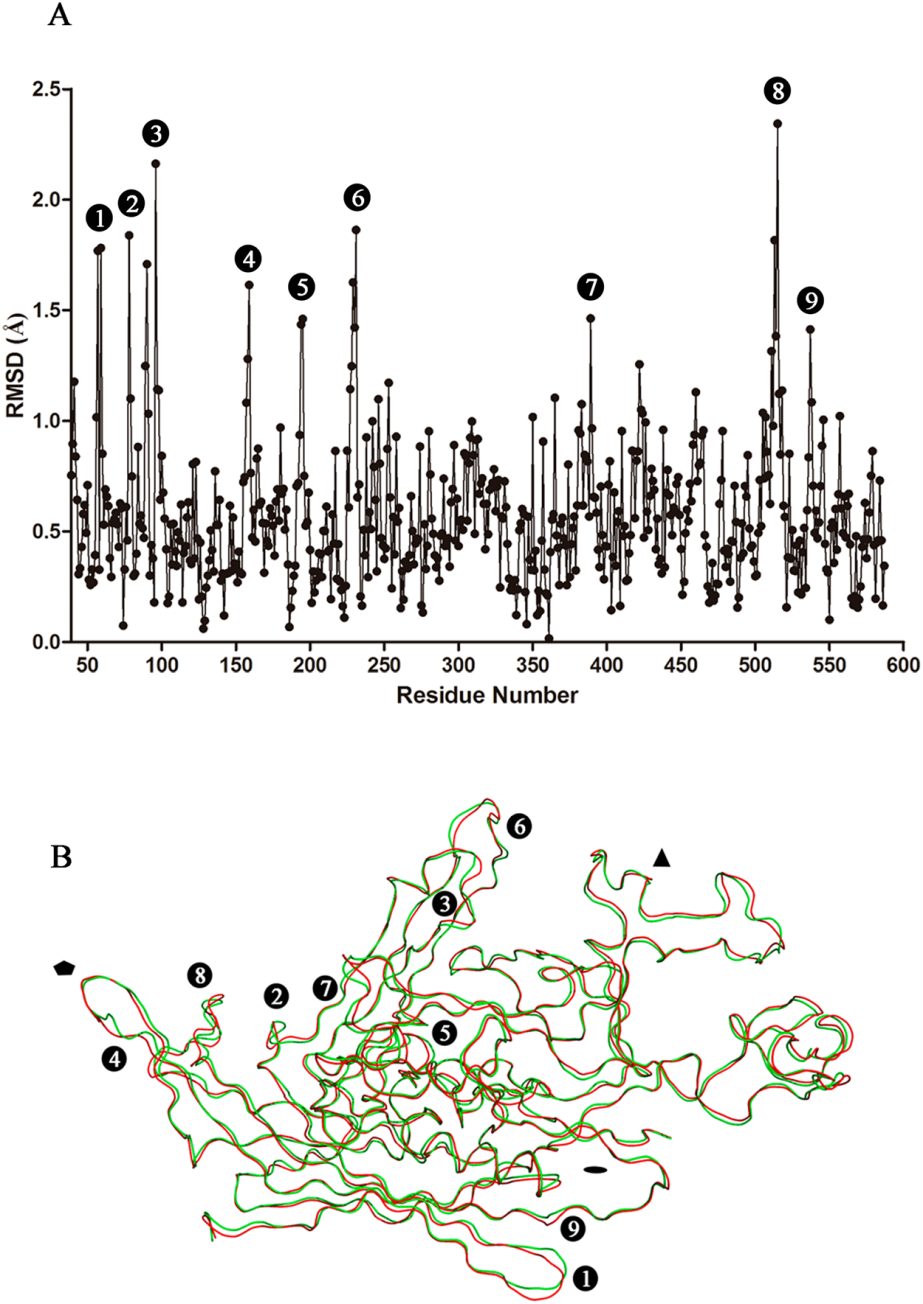
Comparison between the VP2 structure in the wt and mutant N170A MVMp capsids. (**A**) Plot showing the root mean square deviation (RMSD) of the distances between Cα atoms after the superimpositions of all equivalent Cα atoms in the VP2 structures of the MVMp wt and N170A mutant. Regions having Cα > 1.5 Å between wt and mutant are labeled 1 to 9. (**B**) Ribbon representation of the superimposition of the VP2 backbone atoms. Red, wt VP2; green, N170A VP2. Regions with RMSDs > 1.5 Å are labeled from 1 to 9 (compare Table 2). The approximate positions of S5, S3 and S2 axes in the capsid are indicated by a black pentagon, triangle or oval, respectively.

Detailed comparative analyses showed that a number of solvent-exposed capsid regions also exhibited highly significant structural differences between N170A and wt capsids, with RMSD exceeding three times the mean value (Fig. 5A,B; Table S1). These regions included, among others: i) the walls of the S5 pores (residues 150–170) ii) the shoulders (residues 219–238) of the large spikes protruding along the S3 axes; iii) the loop linking residues 382–392 located between the S5 and S2 axes; iv) a highly exposed loop between residues 503–519 that is poorly defined in all MVM virion or capsid structures solved to date^41, 43, 44^.

**Figure 6:**
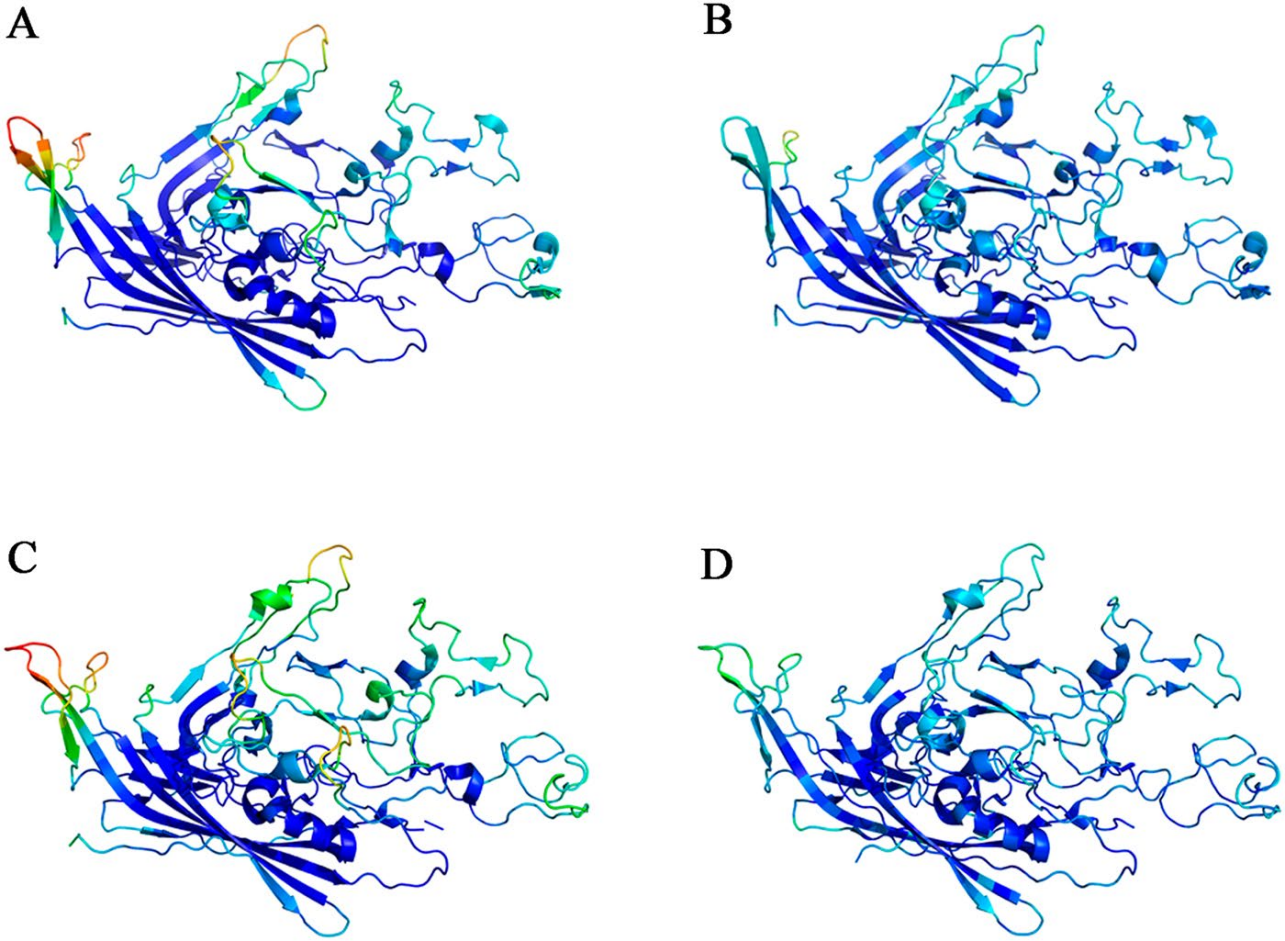
Structure of VP2 in the MVM capsid, color-coded according to normalized B-factors per residue. The color spectrum goes from deep blue (lower value) to bright red (higher value) (**A**) VP2 of wt MVMp (**B**) VP2 of N170A MVMp. (**C**) VP2 of wt MVMi. (**D**) VP2 of L172W MVMi.

Superimposition of the structures of five VP2 subunits around a S5 axis for the wt and N170A MVMp capsids revealed that the subtle changes that occurred in the pore regions as a consequence of the N → A mutation appeared to propagate through the whole capsid, resulting in a more compact overall structure (Supplemental Movie 1). Moreover, comparison of normalized B factors indicated that the four solvent-exposed structural elements mentioned above appear to be highly flexible in the wt capsid crystals, with B factors as much as 4 times above the mean value (Fig. 6A). In contrast, these elements appear to be much less flexible in the N170A capsid crystals, generally exhibiting B factors closer to the mean (except for the 503–519 loop, which was mostly disordered) (Fig. 6B).

In crystals of proteins or protein complexes, including the MVM crystals used in this study, water molecules occupy a large part of the total volume. Protein molecules and MVM particles are surrounded by water molecules and contact each only through packing forces between discrete, small surface regions. Accordingly, it has been generally observed that higher normalized B factors in the crystal correspond to more dynamic structural elements in solution^57^. Importantly, we found that the four MVMp capsid peptide segments with the highest B factors in the crystal correspond quite well with four of the seven segments of the same virus capsid that showed the highest equilibrium dynamics in solution, as determined by hydrogen-deuterium exchange-mass spectrometry (HDX-MS)^56^. The above observations indicate that a single mutation in a viral capsid, N170A in MVM, actually reduces the equilibrium dynamics of several structural elements scattered along the particle, both in the crystal and in solution.

### Compared structural effects of N170A and L172W mutations in the MVM capsid

Like N170A^53^, the L172W mutation at the tightest constriction of the S5 pores (Fig. 1C) is deleterious for MVM at physiological temperature^54^. Plevka *et al*. solved the crystal structure of the L172W mutant of the VP2-only capsid of MVMi at 4.2 Å resolution^44^ (PDB id: 2XGK), and compared it to that of the wt capsid in the MVMi virion at 3.5 Å resolution (PDB id: 1Z1C)^43^. The structure of the wt empty capsid of MVMi was (and is) not available; comparison with the wt capsid of MVMp would not be adequate, because the capsid of strains p and i differ in 13 residues per subunit. Those authors found a high overall structural similarity between L172W and wt capsids of strain i (RMSD of 0.3 Å for equivalent Cα atoms in the 542 residues superimposed); however, conspicuous differences were found in the pores and in Nt organization. Together with phenotypic analysis, their comparison revealed that the ring of tryptophan 172 side chains reduces the diameter of the pore constriction, and sterically prevents viral DNA packaging^44, 54^.

Availability of the L172W capsid structure coordinates allowed us to compare at atomic resolution the changes in the capsid structure of two different MVM strains elicited by two capsid-stiffening mutations located at dynamic functional pores. This comparison was particularly interesting because: i) both N170A and L172W mutations involve neighboring residues lining the capsid pore wall, but the former is a cavity-creating mutation, while the latter is a spacefilling mutation; and ii) both mutations impair virus infectivity, but through a different mechanism. Thus, we have carried out further structural comparisons specifically looking for small conformational differences caused by the L172W mutation that could resemble or oppose those caused by the N170A mutation.

Superimposition of five capsid subunits around a S5 axis in the structures of L172W and wt MVMi capsids revealed a slightly more compact overall structure for the L172W capsid (Supplemental Movie 2). Moreover, parallel comparisons of normalized B factors between capsid structures showed that the four regions that exhibited the largest normalized B values in wt MVMp (see above and Fig. 6A) also exhibited high normalized B values in wt MVMi (Fig. 6C). In contrast, the L172W capsid (Fig. 6D), like the N170A capsid (Fig. 6B), exhibited normalized B values in these regions that were much closer to the mean, suggesting a rigidification of the same structural elements in both mutants. In addition, in the L172W capsid rigidification was also extended to the loop including residues 503–519. Thus, both deleterious, capsid-stiffening mutations at the wall of the capsid pores (cavity-creating N170A and spacefilling L172W) cause a similar, subtle but significant compaction and  drastic rigidification of the MVM capsid structure.

### Structural effects that may underlie mutation-induced mechanical stiffening of a virus particle

When “pushing” with the AFM tip on a protein-made virus capsid, multiple, weak non-covalent interactions (van der Waals contacts, including “hydrophobic” carbon-carbon contacts, hydrogen bonds, coulombic interactions, etc.) between atoms are likely stressed and eventually disrupted by the force applied. Provided the force is not too strong and the indentation is not too deep, the interactions will recover after the AFM tip is retracted. As a result, atoms are reversibly displaced from their original positions, and the force-induced, small, reversible variation in capsid conformation is physically described as an elastic deformation^4, 7, 8, 10, 14^. The resistance of atoms to be displaced may depend on the direction of the force relative to, for example, the orientation of hydrogen bonds, covalent constraints or steric clashes encountered when moving an atom along a particular direction but not others^10^, etc. All-atom MD has predicted mechanical effects of point mutations in small proteins^58^, but not in the much larger virus capsids. AFM analysis have provided some support for a general relationship, for different variants of a same virus particle, between increased number of intraparticle interactions and increased stiffness^18, 24, 30^.

Comparison of atomic structures of the natural MVM capsid and the N170A mutant capsid unveiled unpredicted structural alterations that may explain the general stiffening by mutation N170A (and perhaps also by other cavity-creating mutations around the capsid pores)^19^. Removal of the buried amide group of N170 in each subunit prevented the establishment of local interactions such as hydrogen bonds involving this group, let to the generation of a cavity and some local structural rearrangements, as previously shown for small proteins^59^. But, in addition, the structural effect of this cavity-creating mutation in MVM propagated to the entire capsid, leading to subtle but significant conformational rearrangements of multiple structural elements located far from the mutation site. Most of the rearranged elements, both around the pores and at distant regions, showed substantially reduced normalized B factors in the crystal, indicative of reduced structural flexibility, as discussed above. Together, these rearrangements led to a more compact, less flexible conformation of the N170A mutant capsid in which the overall strength of multiple noncovalent interactions may be increased. The results provide an experiment-based structural explanation for the local and global mechanical stiffening effects observed for a single, cavity-creating, deleterious mutation in a virus capsid.

The above structural interpretation for capsid stiffening is not specific for cavity-creating mutations like N170A. In fact, the spacefilling, deleterious L172W mutation led to similar changes in conformation and rigidification of specific structural elements scattered over the MVM capsid, and to a subtle overall structural compaction of the viral capsid. Many other single mutations at the pores or elsewhere in the MVM capsid also increased capsid stiffness, while virtually no tested mutation led to stiffness decreases^19, 60^. Further structure-mechanics-dynamics studies may ascertain whether the natural MVM capsid has evolved to a state of lowest stiffness compared to close variants in the sequence space, and whether this local minimum in stiffness may be biologically advantageous.

### Biological implications

As summarized and referenced in the Introduction, P. Tattersall, J.M. Almendral and other researchers discovered a critical involvement in the MVM infectious cycle of different translocation events through capsid pores, including genome packaging and uncoating and externalization of VP1 or VP2 Nts. However, the crystal structures of both the wt MVMi virion and wt MVMp capsid revealed that the free diameter of the pore at equilibrium (~8 Å) is not wide enough to allow the passage of the viral polynucleotide during genome packaging and release, or Nt polypeptides during translocation of biological signals^41, 43^. Thus, the pores must be conformationally dynamic, considerably increasing their aperture in response to biological stimuli^46^. Indeed, recent HDX-MS analysis^56^ has revealed that the pore regions are among the most dynamic elements of the MVM capsid in solution at equilibrium, both at low (0 °C) and physiological temperatures.

Several capsid residues located around the base of each pore, including N170 and L172, are critical for MVM infectivity at physiological temperature^53, 54^. Replacement of L172 by several other residues and of N170 (or neighboring residues) by alanine led to multiple phenotypic defects, including either unconditional or temperature-sensitive deficiencies in capsid assembly, genome packaging, uncoating, externalization of Nts and/or entry into cells^46, 53–55^. We discuss here the cases of N170A and L172W mutants for which both capsid mechanical stiffness values and atomic structure are now available.

It must be noted first that the DNA inside the virion stiffens the particle^18, 25^. Thus, any discussion on a possible relationship between stiffness around the pores and virus infectivity would benefit from mechanical analysis of N170A and L172W mutant virions, in addition to capsids. Unfortunately, the deleterious effects of these mutations precluded such analysis. However, some previous experimental observations, mentioned next, lead us to propose that the results obtained here using only empty capsids may be relevant in the context of the DNA-filled virion: i) the virion-stiffening effect of the DNA is specifically due to DNA segments that are bound close to S2 axes, and stiffen the viral particle only locally, i.e., at S2 regions (and to a lower extent, S3 regions located nearby). The presence of the DNA has no effect whatsoever on the stiffness of the S5 axis (pore) regions. Thus, at the regions involved in the biological effects discussed in this study, both virion and empty capsid have the same stiffness; and ii) both the virion and the empty capsid undergo the conformational transition related to pore dynamics and externalization of capsid subunits Nts through the capsid pores.

The severe genome packaging defect of mutation L172W was traced to a reduction in the minimum diameter of the capsid pore (from 8 Å to 6 Å)^44^. It could be argued that the pore, despite its reduced diameter at equilibrium, could still transiently open enough to allow translocation events. However, the remarkable reduction in normalized B factors at the β-cylinder surrounding the pore in the L172W mutant capsid suggests that, in addition to a reduction in diameter, pore dynamics is also substantially reduced, contributing to the observed defect in genome packaging and the lethality of the L172W mutation.

Mutation N170A led, among other effects, to the disappearance of a conformational transition associated to VP2 Nt externalization from the capsid^53^ and to a premature exit of the DNA from the virion^46^. These observations led to the prediction that the free diameter of the pore would be enlarged in the N170A mutant^46^, which could lead to constitutive externalization of VP 2 Nts at physiological temperature. In fact, the crystal structure of the N170A capsid indicates that the N170A mutation: i) does not enlarge, but slightly reduces the pore diameter at equilibrium; ii) substantially reduces pore dynamics, as suggested by the large reduction in normalized B factors at the β-cylinder surrounding the pore, and the mechanical stiffening of the S5 (pore) regions relative to the wt capsid; and iii) does not lead to constitutive externalization of VP2 Nts in the empty capsid, as indicated by three observations: a preserved pore diameter; reduced dynamics and increased stiffness relative to wt; and the absence within the N170A capsid pores of electron density corresponding to the Gly tract located in the VP2 Nt. These results are fully consistent with the proposal that the N170A mutation impairs a capsid conformational rearrangement and associated VP2 Nt externalization^19, 53^, an effect that likely contributes to impaired virus infectivity at physiological temperature^49, 50^.

Interestingly, the spacefilling L172W mutation impaired virus infectivity at physiological temperature, while the isomeric L172I mutation did not^54^. Fluorescence and mechanical analysis (this study) revealed that in the L172W capsid, as in the N170A capsid, the S5 regions were stiffened relative to wt, and the pore-associated conformational transition did not occur. In contrast, in the L172I capsid the S5 regions were not stiffened, and the pore-associated transition was observed^19^. An inextricable linkage between capsid S5 stiffening, loss of this transition and impaired virus infectivity at physiological temperature has been observed also for alanine mutations of other residues at or near the base of the pores^19, 60^. When some of these virus mutants were tested, additional defects, including premature DNA externalization in the virion, were observed, as for the N170A mutant^46^.

Considering the available evidence discussed above, we suggest that the deleterious effect at physiological temperature of the N170A mutation (and, possibly, other cavity-creating mutations around the pores) may depend both on changes in the opening and closing of the pores, and also on some of the observed non-local reductions in capsid structural flexibility and increased mechanical stiffness. For example, the lower flexibility of structural elements and increased stiffness at S2/S3 regions close to the DNA binding sites in the capsid could reduce the affinity of the DNA-capsid interactions observed in the crystal structure of the MVM virion. This effect could facilitate the premature release of the viral DNA by overcoming the opposing effect of conformationally constrained pore regions. Impaired pore dynamics, in turn, would also contribute to reduce infectivity by impairing Nt externalization. Further experiments are required to demonstrate the virus-inactivating mechanism caused by this mutation. Irrespective of the precise mechanism, the present results reinforce the evidence for a complex relationship in a virus capsid between structural compactness and mechanical stiffness, reduced conformational dynamics and impaired biological function.

### Nanotechnological and biomedical implications

The relatively high mechanical elasticity of many virus particles and their associated propensity to undergo conformational fluctuations and transitions may make them inadequate for use as nanoparticles in a number of applications^39^. For example, in cases where a precise distance between attached components is required^37^, or leaching of confined cargo molecules through dynamic capsid pores must be prevented^61^. Rational protein engineering of virus-based nanoparticles by introducing mutations with compaction-inducing effects similar to that of N170A in the MVM capsid may increase their rigidity and improve their suitability for such applications.

This study encourages also the exploration of novel antiviral drugs favoring a more compact conformational state of the virus particle. A compound-induced overall compaction of a viral particle, similar to the effect of the N170A in the MVM capsid, could reduce the local or global conformational dynamics required during the infectious cycle of many viruses^62, 63^. AFM could be used as a rapid and straightforward technique to detect, under close to physiological conditions, the effect of different compounds on viral particle stiffness, as an indicator of altered conformational dynamics^19, 20^. We have already provided proof-of-concept that inverse variations in conformational dynamics and stiffness of the human immunodeficiency virus capsid lattice can be achieved by addition of a small molecule (betaine), and readily analyzed by AFM^64^.

## Conclusion

Mechanical analysis by AFM revealed that both cavity-creating (N170A) and spacefilling (L172W) deleterious mutations of amino acid residues that delineate functional pores in the MVM capsid cause large local and global increases in mechanical stiffness. X-ray structure determination of the N170A mutant capsid and comparison with the crystal structure of the non-mutated capsid revealed subtle conformational differences that propagate through the whole capsid, resulting in a more compact and less flexible structure in the mutant. The same relationship between increased global structural compaction, reduced conformational flexibility and increased mechanical stiffness was found for the L172W mutation. The results reveal a structural basis for the biologically relevant rigidification independently caused by a cavity-creating mutation and a spacefilling mutation in a virus particle.

## Methods

### Recombinant plasmids and mutagenesis

Plasmid pMVMiΔVP1 was kindly provided by Prof. J.M. Almendral (Centro de Biología Molecular “Severo Ochoa”, Madrid, Spain)^65^. The VP2-coding region of MVMi contained in pMVMiΔVP1 was amplified by PCR, and restriction sites BamHI and XhoI were used to subclone this region into a pFastBac1 vector (Invitrogen) as previously described^52^, to yield plasmid pFB1-VP2i. Site-directed mutagenesis was carried out on pFastBac1 using the QuikChange kit (Stratagene), and the presence of the introduced mutation was confirmed by DNA sequencing. pFB1-VP2i (wt and mutant L172W) were used to construct the baculovirus shuttle vectors (bacmids BM-VP2i) containing the corresponding VP2 genes of MVMi (wt and mutant L172W). The construction of BM-VP2p bacmids containing the VP2 gene of MVMp (wt and mutant N170A) has been previously described^52, 53^.

### Expression and purification of MVM capsids

VP2-only capsids of MVMp and MVMi (wts and mutants N170A and L172W) were produced in H5 insect cells using a recombinant baculovirus-based expression system and BM-VP2p and BM-VP2i bacmids (wts and mutants) as previously described^19^. Capsid purification was carried out essentially as previously described^42, 48^. Purity and quality of the capsid preparations was assessed by electron microscopy.

### Crystallization, data collection and processing

Crystals of MVMp capsid mutant N170A were obtained using the hanging drop vapour diffusion method at 20 °C. Briefly, 1 μl of purified capsid solution (10 mg/ml) in 10 mM Tris-HCl, pH 7.5, was mixed with an equal volume of reservoir solution containing 14% 2-methyl-2,4-pentanediol (MPD), 200 mM NaCl, 100 mM NaAc, pH = 4.5. Crystals formed in 3–4 days. For data collection crystals were incubated for 1 min. in reservoir solution plus 20% MPD as cryo-protectant and flash-frozen under liquid nitrogen. X-ray data were collected at the European Synchrotron Radiation Facility, Grenoble, France (beam line ID23–1). Diffraction images from one single crystal were processed using XDS^66^ and SCALA^67^.

### Structure solution and refinement

Crystals belong to the rhombohedral space group R32 with cell parameters of a = b = 410.24 Å; c = 559.77 Å. The packing considerations indicated that there were two particles per unit cell, with two independent decamers of a virus particle occupying a crystallographic asymmetric unit. Initial phases were determined by molecular replacement with the program PHASER^68^ using the crystal structure of the VP2-only empty capsid of wt MVMp (PDB id: 1Z14)^43^ as a searching model. After several cycles of rigid body refinement using REFMAC5^69^ and manual rebuilding with COOT^70^, refinement including 20-fold non-crystallographic symmetry constraints was performed in CNS^71^ until the model reached R_work_ and R_free_ of 27.6 and 28.1, respectively. Cycles of 20-fold non-crystallographic averaging and solvent flattening with the DM program^72^ were applied to improve map quality. Averaging and solvent masks were created using NcsMask^73^. Statistics for both data collection and refinement are summarized in Table 1. Normalized B factors were calculated by dividing the B factor values of the individual residues by the average B factor calculated for all atoms. Atomic coordinates of the N170A MVMp capsid have been deposited in the PDB (id: 4ZPY).

### Atomic force microscopy

AFM hardware and software was from Nanotec Electrónica, S.L. AFM imaging and determination of the mechanical stiffness of individual wild-type or mutant MVM capsids was carried out essentially as described^18, 25^. Briefly, a drop of the sample (∼20 μl) in phosphate-buffered saline (PBS) was deposited onto a hexamethyldisilazane (Sigma-Aldrich)-treated glass coverslip and left for 30 min. at room temperature for capsid adsorption. AFM images were obtained in Jumping Mode^74^ using RC800PSA cantilevers (Olympus) with a nominal spring constant of 0.1 N/m, and keeping the force applied under ∼50 pN. Before starting each experiment, the actual spring constant *k*_c_ of each cantilever used was calibrated using Sader’s method^75^. AFM images were processed using WSxM software^76^. The mechanical stiffness of different regions in the MVM capsid was determined by indenting a number of single capsids of known orientation with a 5-fold, 3-fold or 2-fold symmetry axis close to the top of the particle at the indentation point (Figs S2 and S3 of SI)^25^. To keep the conditions within the range of a linear elastic response and avoid particle damage, only force-versus-deformation (F-D) measurements that involved indentations between 0.5 nm and 2.0 nm were considered. The local stiffness of each capsid region (around a 5-fold, 3-fold or a 2-fold axis) was determined by calculating the corresponding spring constant *k*_s_, assuming that viral particle and AFM cantilever behave as two ideal springs in series^14, 18, 25^.

### Statistical analysis of mechanical data

The *k*_s_ values obtained for MVM capsids followed a normal distribution described by a Gaussian fitting, as corroborated using normality tests (and as observed for other viral particles). The statistical significance of differences in *k*_s_ values between mutant and parent capsids was assessed using OriginPro8 (OriginLab). To statistically validate that two *k*_s_ are different, the two-population two-tailed Student t-test was used, with an alpha level of 0.05. Equal variance was not assumed, providing a more stringent test.

## Electronic supplementary material

Video 1

Video 2

Supplementary Figures and Table
