# Supplementary material for: Structural basis for biologically relevant mechanical stiffening of a virus capsid by cavity-creating or spacefilling mutations
Source: Sci Rep. 2017 Jun 22;7:4101. doi: 10.1038/s41598-017-04345-w (PMC5481337; doi:10.1038/s41598-017-04345-w)
Supplement: Supplementary file 3 — Supplementary Figures and Table [file 41598_2017_4345_MOESM3_ESM.pdf]

SUPPLEMENTARY INFORMATION

Structural basis for biologically relevant mechanical stiffening of a virus capsid by cavity-creating or spacefilling mutations

Pablo Guerra,[1] Alejandro Valbuena,[2] Jordi Querol,[1] Cristina Silva,[1] Milagros Castellanos,[2] Alicia Rodríguez-Huete,[2] Damià Garriga,[1‡] Mauricio G. Mateu,[2,*] and Nuria Verdaguer[1,*]

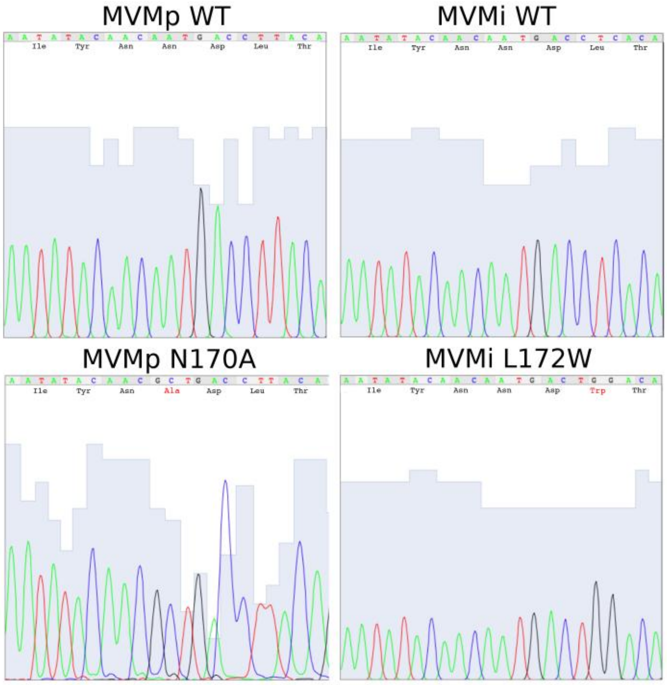

Figure S1. Sequence chromatograms for the part of the MVM capsid region where the introduced mutations are located. left: MVM strain p, wt (top) and N170A mutant (bottom); right: MVM strain i, wt (top) and L172W mutant (bottom).

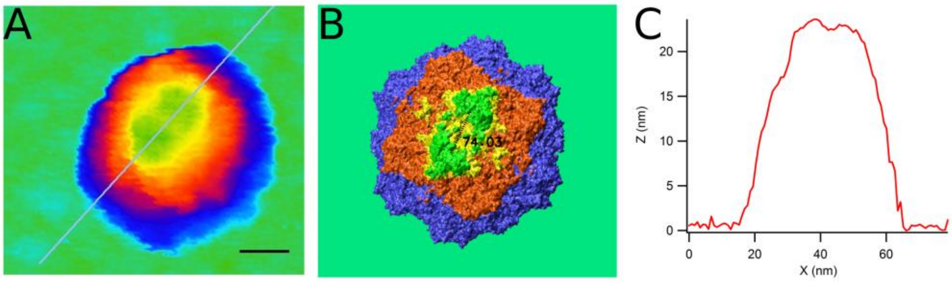

Figure S2. Comparison of the topographic structure of the MVM capsid as determined by AFM or by X-ray crystallography. (A) AFM image of a capsid oriented with a S2 symmetry axis on top. The blue line corresponds to the Z-profile presented in panel C. (B) surface model of the atomic structure of the capsid determined by X-ray crystallography (PDB code 1Z14). In panels A and B, a similar color code (blue-red-yellow-green) has been used to indicate increasing height of different regions in the capsid. In both the AFM image and the crystallographic model, the two green regions on top of the particle correspond to two S3 prominences (spikes) related by a S2 symmetry axis. (C) Z-profile of the particle shown in panel A along the blue line that crosses the particle. Particle height (24 nm) and distance between S3 spikes related by a S2 axis (8.5 nm in the AFM image *versus* 7.4 nm in the crystallographic model) match quite well those determined using the atomic model.

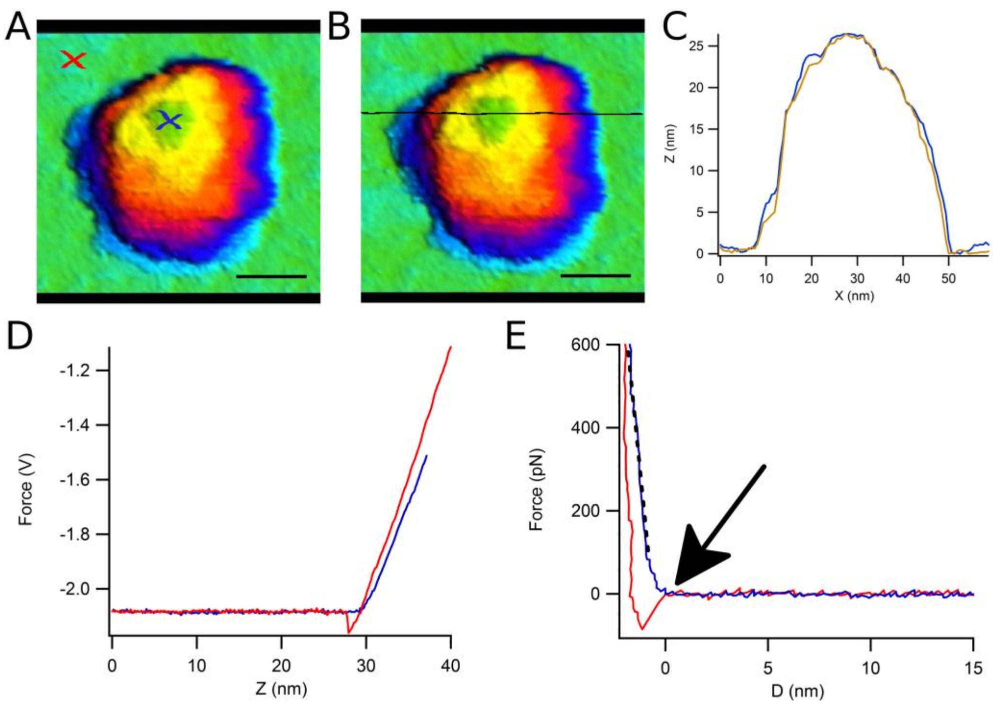

Figure S3. Procedure followed to determine elastic constants of viral particles by AFM[14]. (A,B) AFM images of the particle before (A) or after (B) indentation with the AFM tip (in this case on the region around a S3 axis (colored green) in a MVM capsid. Scale bar corresponds to 15 nm. Image comparison shows that the viral particle has not been reoriented during indentation. Scale bars are 15 nm in length. (C) height profiles of the particle before (blue) and after (orange) indentation. The black horizontal line in panel B indicates the pathway followed to obtain both height profiles. The profiles are coincident, which shows that, after being indented, the particle fully recovers its original height. Vertical resolution is < 1nm. (D) representative F-Z plots corresponding to force (expressed in volts) *versus* piezoelectric displacement along the Z axis (nm). The red trace was obtained by pushing on the substrate (red cross on the image in panel A), and the blue trace was obtained by pushing on top of the particle (blue cross on the image in panel B. (E) F-D plots obtained by converting the F-Z data shown in panel D, and corresponding to force (in pN) *versus* indentation (particle deformation) (in nm). Color code to represent F-D curves corresponds to that used to represent F-Z curves in panel D. The black dashed line delimits the linear region used to calculate the spring constant of the particle. The arrow indicates the contact point.

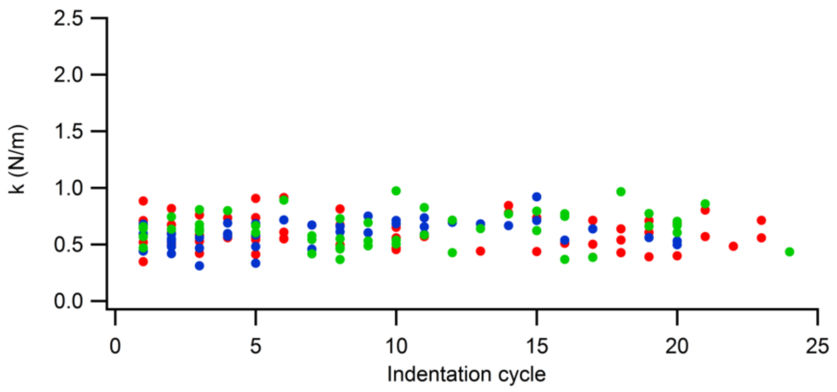

Figure S4. Elastic constant values obtained on a same capsid (wt MVMp) as a function of the number of indentation cycle. Red, blue and green dots respectively correspond to values obtained by indentation at a S2, S3 or S5 axis.

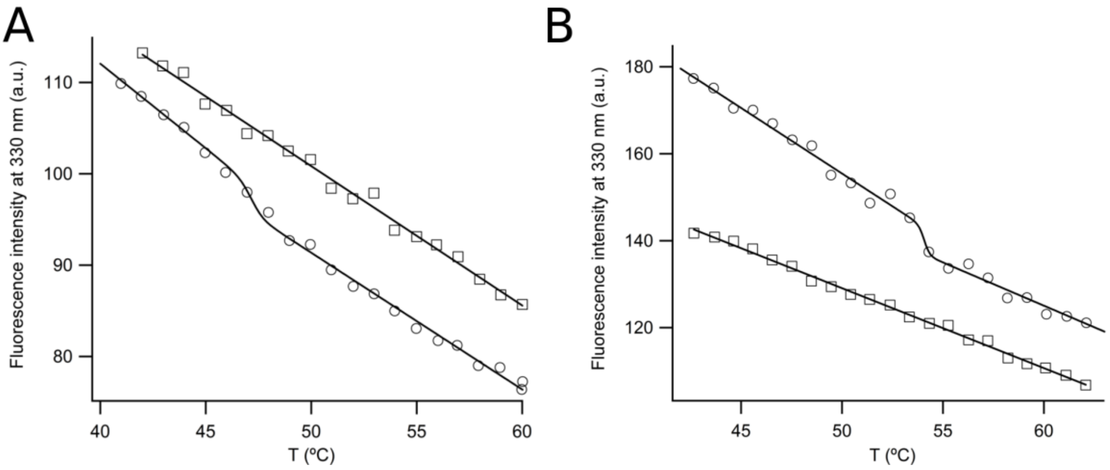

Figure S5. Intrinsic Trp fluorescence intensity at 330 nm of MVMp (A) and MVMi (B) capsids as a function of temperature. Circles, strain p (A) or strain i (B) wt capsid; squares, N170A (A) or L172W (B) mutant capsid. a.u., arbitrary units.

Table S1. VP2 regions exhibiting RMSD differences exceeding three times the mean value when the structures of wt and mutant N170A MVMp capsids were compared

Region	Capsid location	Residues
1	Bottom of S2 axes	55 - 60
2	Floor of depression close to S5 axes	76 - 81
3	Shoulder of S3 protrusion	88 - 101
4	Top of S5 axes	156 - 164
5	Floor of depression between S3 and S5 axes	192 - 196
6	Shoulder of S3 protrusion	225 - 233
7	Wall between S5 and S2 axes	380 - 391
8	Floor of depression close to S5 axes	510 - 519
9	Bottom of S2 axes	536 - 540

Movie S1. Superimposition of the structures (cartoon model) of five VP2 subunits around a S5 axis for the MVMp wt and N170A capsids. The red segment indicates the location of the Cα of residue 170. The animation proceeds from the N170A mutant structure to the wt structure.

Movie S2. Superimposition of the structures (cartoon model) of five VP2 subunits around a S5 axis for the MVMi wt and L172W capsids. The red segment indicates the location of the Cα of residue 172. The animation proceeds from the L172W mutant structure to the wt mutant structure.
